# Four-Digit Replantation in a Mentally Retarded Person: A Case Report

**Published:** 2010-09-30

**Authors:** N. Sinis, M. Boettcher, A. Kraus, F. Werdin, H. E. Schaller

**Affiliations:** ^a^Department for Plastic and Reconstructive Surgery with Hand Surgery, Marthin-Luther Hospital, Berlin; ^b^Department for Hand, Plastic, Reconstructive Surgery With Burn Unit, University of Tuebingen, Tuebingen, Germany.

## Abstract

This report describes a case where 4 digits were replanted in a mentally retarded patient with a history of smoking and the inability to follow postoperative arrangements.

With the advent of microsurgical techniques, digital and hand replantation has become a life-enhancing surgical procedure. Indications for replantation have evolved over the years and currently include hand amputation, thumb amputations, multiple digit amputations, and amputations in children.^1^ However, crush and avulsion injuries and amputations of a single digit proximal to the superficial tendon of the flexor digitorum muscle insertion remain controversial.^2^ Even though replantation surgery has now become a routine procedure, it remains a delicate and demanding surgery, requiring adequate training and expertise in microsurgical techniques.^1^,^3^

Overall, experience demonstrates that not all amputees will benefit from, or are candidates for, replantation. The decision to proceed should therefore be made by the surgeon only after assessing the mechanism and extent of injury, absence of relative contraindication such as smoking, the likely functional outcomes, and the patient's motivation to undergo a difficult procedure, which is followed by a lengthy recovery.^3^ However, considering the patient's specific demands will ensure satisfaction that often outweighs even poor functional outcomes.

In the following case, we replanted 4 digits in a mentally retarded patient with a history of smoking and the inability to follow postoperative arrangements. To grant the patient's wish to keep his fingers, he was sedated for 2 weeks postoperatively, creating a very compromising situation.

## CASE REPORT

### History of present illness

On January 29, 2008, a 31-year-old mentally retarded man sustained a complete amputation of his right index, middle, ring, and small fingers by slipping from a shelf into a buzz saw. The patient was admitted 2 hours later to the emergency department of our hospital. The 4 fingers had been wrapped with gauze and properly preserved in a bag of icy water. Replantation procedure started 3 hours after amputation injury under general anesthesia.

### Physical examination at first presentation

**Figure 1:**
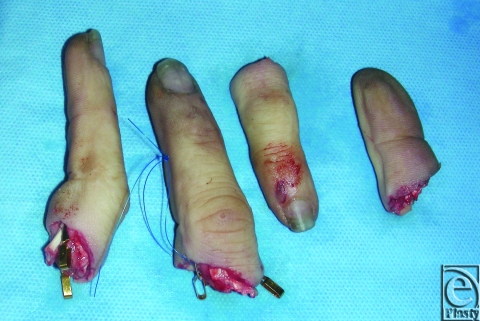

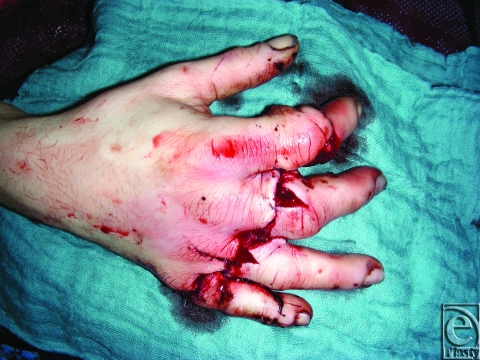
Amputated digits (a) and immediate postoperative view on replanted digits (b).

The 4 fingers were completely amputated through various levels of the proximal phalanges, leaving the metacarpophalangeal joints intact (see Figure 1a). The pattern of injury was:

digit II—amputation at the proximal interphalangeal joint with destruction of the joint.
digit III—amputation at the distal level of the proximal phalanges.
digit IV—amputation at the middle level of the proximal phalanges.
digit V—amputation at the proximal level of the proximal phalanges.

### Surgical intervention

The patient was operated under general anesthesia without a tourniquet. After intraoperative x-ray evaluation, the bones were stabilized by shortening (proximal and distal to the amputation line with about 3 to 6 mm). After obtaining almost straight bone lines, a wire was used (1 mm) in an oblique fashion with a cerclage to adjust and fix the bones in anatomical position. In contrast to the authors' habits, but considering the involvement of all digits, one artery was sutured at each digit and rinsed with a bolus of 100-mg acetylsalycilacid. In case of a single-finger replantation, the author prefers to continue with the tendon repair, and afterward he performs all the microsurgical steps. After ensuring perfusion of all digits the second artery was sutured at every digit (see Figure 1b). On digits II, III, and IV, 2 veins were sutured. On digit V only, one vein was anastomosed. The extensor and superficial as well as deep flexor tendons were sutured with nonresorbable sutures. Finally, the 2 palmar nerves of each digit were sewed with a microsuture. After subtile hemostasis using bipolar coagulator, the skin was closed loosely. A soft dressing was applied and the patient was extubated after 12 hours' operating time.

### Postoperative course

After surgery, the patient was kept in a warm room to promote vasodilation for 5 days. Hematocrit, hemoglobin, blood pressure, and electrolyte balance were carefully monitored. We started the patient on low-molecular-weight dextran (Dextran-40) and kept him anticoaguated (100 mg aspirin and 10.000 units of low-molecular-weight heparin via continuous intravenous application daily) for 10 days. Hourly monitoring with pulse-oximetry and capillary refill was conducted until postoperative day 14. To avoid unintentional injury by the mentally retarded patient, a sedative drug (100 mg Melperone daily) was applied intravenously. Due to a thrombosis of the vein in the fifth digit the patient was retaken to the operating room after 46 hours. To restore sufficient venous outflow an additional vein was anastomosed. Unfortunately, the fifth digit demarcated in the following days and had to be amputated with exarticulation in the metacarpophalangeal joint. On day 15, sedation was stopped, the hand was protected in a circular plaster for 4 weeks, and twice daily passive mobilization was started. Finally, the patient was discharged 6 weeks after the operation.

**Figure:**
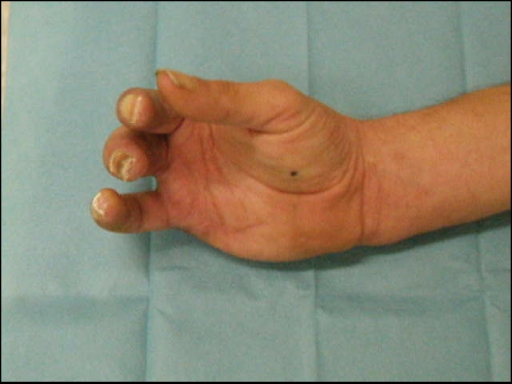
“This video is in QuickTime format. If you do not have QuickTime you may download it here”.

**Figure 2:**
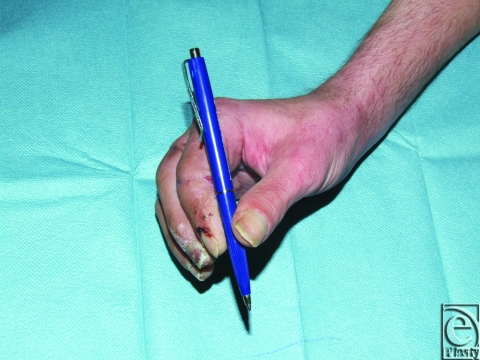

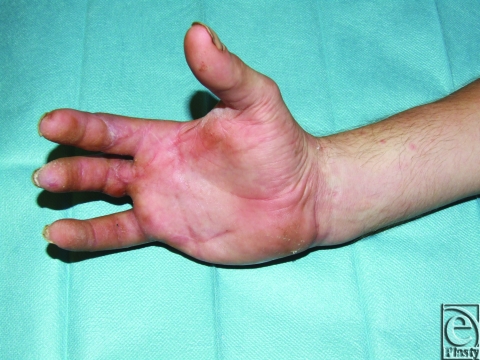

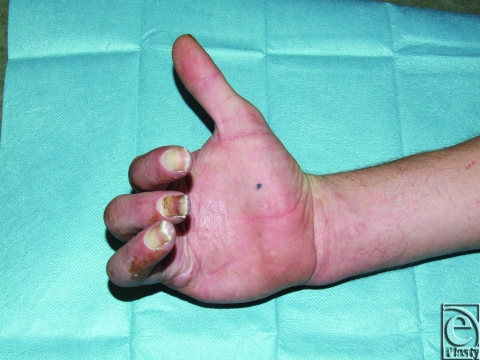

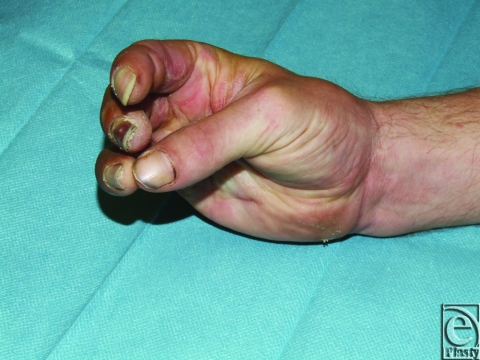
(a) Long-term functional result after reamputation of the fifth digit. Note the patient holding a pen to write. (b) One year after operation, the patient demonstrates maximum of extension in the digits. The distance between thumb and fourth digit was 12 cm at the tip. (c) One year after operation, the maximum of finger flexion is depicted. (d) One year after the operation, the patient was able to reach all tips of the replanted fingers with his thumb.

One year postsurgery the patient was capable of grasping small and big objects with his hands (M5). However, function of the digits was impaired. The patient demonstrated an incomplete fist with a distance from the tip of the digits II to IV into the palm of 2, 2, and 3 cm. The main function was observed in the metacarpophalangeal joints with an excursion of 50° from a neutral joint position (ie, 0 degree) for flexion. The middle and distal interphalaneal joints were without function and mainly stiff. Nevertheless, because of a stiffness in a flexed position in the middle interphalangeal joints (about 60° for all) grasping was facilitated. He was able to write and distinguish sharp from blunt stimulus with digits II, III, and IV (see Figure 2a–2d).

## DISCUSSION

Despite the fact that functional outcome of replanted hands and fingers will never equal that of the healthy counterpart, replantation has major functional and psychological benefits.^4^ Especially restoration of function in multiple digit amputation is quite decisive for daily activities. Many authors found that multiple finger amputations are a strong indication for replantation.^1^,^3^,^5^ When considering multiple-finger replantation, the finger with the best chance for successful replantation and most significant contribution to function should be repaired first. If all the fingers are injured equally and have the same chance for successful repair, many surgeons prefer to repair the middle, then index, then ring, and, lastly, the small finger.^1^,^5^ Patient satisfaction hinges on their level of expectation as defined and explained in the preoperative discussion and informed consent process.^5^ However, difficulties arise when the patients wish for replantation but existing preconditions or contraindications make it difficult to satisfy the demands.

The presented case is a rare example of a mentally disabled patient with replantation of 4 fingers who clearly wished to save all digits. Despite the possibility of self-inflicted but not intentional injuries to the amputees and a history of heavy smoking, the authors decided to grant the patient's wish. The responsible legal guardian supported the strategy and gave written informed consent to replantation and postoperative sedation. This management is not without risk. Melperone is an antipsychotic drug that has been reported to have atypical properties, that is, low extrapyramidal side effect. It may cause serious cardiovascular side effects, including prolonged QT interval, eventually leading to torsades de pointes and sudden death.^6^ In addition, long-term postoperative sedation comes with an increased risk of falling and injury.^7^

The authors believe that in this current case sedation was the only option to guarantee the patient's compliance and to preserve the postoperative results.

At the 1-year follow-up, patient and guardian told us that they were very happy with the result and that they would do it again.

## CONCLUSION

Multiple finger replantation is a challenging microsurgical procedure made even more difficult in noncompliant patients. The presented case report demonstrates one possible solution to achieve optimal postoperative management in an incompliant patient with quite impressive results.

## ACKNOWLEDGMENTS

No author has any conflict of interest with the presented data.
